# Metacognitive Abilities as a Protective Factor for the Occurrence of Psychotic-Like Experiences in a Non-clinical Population

**DOI:** 10.3389/fpsyg.2022.805435

**Published:** 2022-02-24

**Authors:** Marco Giugliano, Claudio Contrada, Ludovica Foglia, Francesca Francese, Roberta Romano, Marilena Dello Iacono, Eleonora Di Fausto, Mariateresa Esposito, Carla Azzara, Elena Bilotta, Antonino Carcione, Giuseppe Nicolò

**Affiliations:** ^1^Società Italiana di Cognitivismo Clinico, Rome, Italy; ^2^Terzo Centro di Psicoterapia, Rome, Italy

**Keywords:** psychotic-like experiences, metacognition, delusions, hallucinations, Metacognition Self-Assessment Scale, psychopathology

## Abstract

Psychotic-like experiences (PLEs) are a phenomenon that occurs in the general population experiencing delusional thoughts and hallucinations without being in a clinical condition. PLEs involve erroneous attributions of inner cognitive events to the external environment and the presence of intrusive thoughts influenced by dysfunctional beliefs; for these reasons, the role played by metacognition has been largely studied. This study investigates PLEs in a non-clinical population and discriminating factors involved in this kind of experience, among which metacognition, as well as psychopathological features, seems to have a crucial role. The aim of this study was to extend the knowledge about the relationship between metacognition, psychopathology, and PLEs, orienting the focus on metacognitive functioning. The sample consisted of 207 Italian participants (men = 32% and women = 68%) voluntarily recruited online, who gave consent to participate in the study. The average age of the sample was 32.69 years (SD: 9.63; range: 18–71). Subjects affected by psychosis, neurological disease, and drug addiction were excluded from the analyses. The following scales were used to investigate PLEs: Peters et al. Delusions Inventory (PDI), Launay-Slade Hallucinations Scale-Extended Revised (LSHSE), Prodromal Questionnaire-Brief (PQ-B), and Revised Hallucination Scale (RHS). To assess general psychopathological features, the Behavior and Symptom Identification Scale (BASIS-32) was administrated. The Metacognition Self-Assessment Scale (MSAS) was chosen to evaluate metacognitive functioning. From hierarchical regression analyses, it emerged that the presence of anxiety, depression, and impulsive/addictive symptoms constitute a remarkable vulnerability factor for PLEs, in line with previous evidence regarding the relationship between general psychopathology and PLEs. Metacognition negatively predicts PLEs, and its presence does not affect the significance of psychopathological variables, suggesting that metacognitive abilities seem to play a protective role for the occurrence of PLEs among non-clinical individuals, and such ability operates as an independent predictor along with other variables. These results are explained by the role of metacognitive functions, which allow individuals to operate many mental processes such as interpreting sensorial events as real or illusory, understanding behaviors, thoughts, and drives of others, and questioning the subjective interpretation of facts.

## Introduction

Psychotic-like experiences (PLEs) are a phenomenon that occurs in the general population experiencing delusional thoughts and hallucinations without being in a clinical condition (Kelleher and Cannon, 2011). Delusional thoughts are commonly experienced by the subclinical population (Heilskov et al., 2020) as well as hallucinations (Larøi et al., 2019), and recently, many studies suggested that between 5 and 7% of adults incur PLEs during their lifetimes (van Os et al., 2009; McGrath et al., 2015; Maijer et al., 2018; Healy and Cannon, 2020). Johns and Van Os reviewed the occurrence of psychotic symptoms within the general population (between 5 and 8%) and proposed an extended psychosis phenotype, suggesting that symptoms reported by the non-clinical population and symptoms reported by patients lie on the same multidimensional continuum (Johns and van Os, 2001), with non-psychotic individuals that may have less severe experiences compared with psychotic individuals and, moreover, better reality testing in the absence of clinical levels of distress or functional impairment. PLEs involve erroneous attributions of inner cognitive events to the external environment (Hoffman, 1986; Bentall, 1990a,b; Frith, 1992; Larøi and Woodward, 2007) and erroneous attributions of intrusive thoughts influenced by dysfunctional beliefs (Morrison et al., 1995), and for these reasons, the role played by metacognition was largely studied. Most of the publications on this topic refer to the self-regulatory executive function model (S-REF; Wells and Matthews, 1996), which defines metacognition as “the aspect of information processing that monitors, interprets, evaluates, and regulates the contents and processes of its organization” (Wells and Purdon, 1999). Studies using this perspective show that metacognitive beliefs involving worry and intrusive thoughts promote and maintain delusional and hallucinatory experiences in clinical and non-clinical populations (Larøi and Van Der Linden, 2005; García-Montes et al., 2006; Barkus et al., 2010). S-REF focuses on “thinking about thinking” declined into worry and rumination outcomes, this construct of metacognition is assessed with *Metacognitive Questionnaire* (MCQ; Cartwright-Hatton and Wells, 1997; Wells and Cartwright-Hatton, 2004), and this instrument is oriented to evaluate mental contents rather than mental functions (Faustino et al., 2021). *The Metacognitive Multi-Function Model* (MMFM; Semerari et al., 2003) intends metacognition as “the whole set of abilities that allows us to understand mental phenomena and work them out in order to tackle tasks and master mental states that are a source of subjective sufferance” (Carcione et al., 1997; Carcione and Falcone, 1999). This perspective considers a set of abilities that are crucial to (1) identify mental states and ascribe them to oneself and others based on facial expressions, somatic states, behaviors, and actions; (2) reflect and reason on mental states; and (3) use information about mental states to make decisions, solve problems or psychological and interpersonal conflicts, and cope with subjective suffering (Semerari et al., 2003; Carcione et al., 2019). In addition, the model identifies different metacognitive functions as follows: *monitoring* is the ability to detect emotion and thoughts forming mental states, *integration* is the ability to reflect on mental states and processes due to sorting them in a hierarchy of importance, which permit individuals to behave coherently with their own purposes, *differentiation* is the ability to differentiate between different classes of representation (e.g., dreams, fantasies, and beliefs) and between representations and reality, recognizing their subjectivity, and *decentration* is the ability to define mental states of others by forming hypothesis and *mastery* that is the use of psychological information to cope with problems of different levels of complexity. In comparison with the model suggested by Wells and Matthews, authors keep the subdivision into monitoring and regulating abilities and concentrate on the functional ability to perform certain operations, rather than on the contents (Faustino et al., 2021). Another important point about PLEs is their relationship with general psychopathology. Studies found a link between PLEs and PTSD (Bak et al., 2005; Scott et al., 2007), drug abuse/addiction (Mitchell and Vierkant, 1991; Rössler et al., 2007; Brewer and Collins, 2014), anxiety, and depression (Johns et al., 2004). Regarding metacognition, its interplay with PLEs and general psychopathology as the isolated factor is difficult to assess using MCQ (Brett et al., 2009) as the items of the scale represent peculiar psychopathological impairments particularly related to anxious and depressive symptomatology (Wells et al., 1997), and this can in part explain why there are no findings in the literature about the interplay between these factors. By the way, since MCQ does not allow testing metacognition decoupled from some psychopathological symptoms and there are many studies in support of correlation between PLEs and psychopathological features (Freeman and Fowler, 2009; Armando et al., 2010; Kelleher and Cannon, 2011; Varghese et al., 2011), the role of metacognition in the occurrence of PLEs is unclear. However, the hypothesis of previous studies is probably correct given that metacognitive contents provided in MCQ test the ability to provide mental processes allowing individuals to evaluate their own thoughts and thoughts of others as well as internal or external events. Therefore, the MMFM model understands metacognition in a similar way to the MCQ model but by implementing the construct with other characteristics and through a different scale (Metacognition Self-Assessment Scale, MSAS) which defines metacognition as a set of skills that do not overlap with any psychopathological symptom. Therefore the hypothesis of the present work is that the metacognitive functions, measured with MSAS, can play a role in the PLE as well as in the metacognitive contents and the interaction with psychopathology can be verified given the nature of this model.

In view of the above, the aim of this study was to investigate both PLEs in non-clinical population and discriminating factors involved in these kinds of experiences, among which metacognition seems to have an important role. Since MCQ seems to not discriminate for the evaluation of metacognitive functioning, the MSAS (Pedone et al., 2017) was chosen to assess metacognitive functioning instead of metacognitive contents in order to identify the link between metacognition, psychopathology, and PLEs. The involvement of all the metacognitive functions is expected due to the heterogeneity of PLEs.

## Methods

### Participants and Procedure

The sample consisted of 215 Italian participants (men = 32% and women = 68%) voluntarily recruited online, who gave consent to participate in the study. The average age of the sample was 32.69 years (SD: 9.63; range: 18–71). Education and professional demographics were also measured. As for education, 5.8% did not have a high school diploma, 30.9% had a high school degree, and 63.3% had a college degree. Students comprised 17.2% of the sample, while professionals were 31.2%, white-collar employees 38.1, housewives 3.3%, unemployed 5.1, and 1.4 retired seniors. Finally, 16.9% were currently married, and 27.5% were divorced or separated. Respondents affected by psychosis, neurological disease, and drug addiction were excluded from the analyses (*n* = 8) leaving the sample to 207.

The sample was recruited online during the months of May–July 2019 and was asked to voluntarily participate in the research about unusual experiences and wellbeing. Ten researchers advertised in their social network pages a link to voluntarily participate in the research, described as an inquiry about unusual experiences in everyday life. Every participant was informed about the anonymity of the study and gave consent to participate in the inquiry. The questionnaire took ~20 min to be filled in.

The obtained sample size guaranteed 0.80 power for *r*s as low as 0.19.

### Measures

#### Unusual Experience Scales

**(1) Peters et al. Delusions Inventory**

This is a 21-item Italian version of the *Peters et al. Delusions Inventory* (PDI) (Peters et al., 1999; Preti et al., 2007). The participant was required to rate the degree of distress, preoccupation, and conviction about delusional thoughts (e.g., “Do you ever feel as if you are being persecuted in some way?”) on 5-point Likert scales (1–5) for each positively endorsed item. This scale is typically used to assess delusional ideation in the general population. The scale showed good reliability (α = 0.90).

(2) **Launay-Slade Hallucinations Scale-Extended Revised**

The Italian version of Launay-Slade Hallucinations Scale-Extended Revised (LSHS-E) (Launay and Slade, 1981; Larøi et al., 2004; Larøi and Van Der Linden, 2005; Vellante et al., 2012) is a self-report scale, which investigates the hallucinatory experiences in every sensory modality in the general population (e.g., “I have been troubled by hearing voices in my head”). Subjects have to rate each item on a five-point scale: (0) “certainly does not apply to me”; (1) “possibly does not apply to me”; (2) “unsure”; (3) “possibly applies to me”; and (4) “certainly applies to me.” The time interval considered for the appearance of these experiences is 5 years. The scale showed good reliability (α = 0.86).

**(3) Prodromal Questionnaire-Brief**

This is the Italian version of the Prodromal Questionnaire-Brief (PQ-B) (Preti et al., 2018), which is a yes/no 21-item self-report questionnaire used to assess positive symptoms experienced in the past month in the general population. For each symptom, responders have to rate the level of distress and its related impairment in everyday life, in a range from 1 (strongly disagree) to 5 (strongly agree), with 4 or 5 indicating distress. The total distress score (range: 0–105) is obtained by summing up each item. The scale showed acceptable reliability (α = 0.79).

**(4) Revised Hallucination Scale**

The Revised Hallucination Scale (RHS) is a 24-item questionnaire revised from Launay and Slade (1981) and Morrison et al. (2002). This version incorporates additional items measuring predisposition to auditory and visual hallucinations, vividness of imagery, and daydreaming. Items are endorsed with a 4-point scale measuring frequency. To date, an Italian validation of this instrument does not exist so a mother tongue translator independently translated the items, and international translation practices were employed (Beaton et al., 2002). The scale showed acceptable reliability (α = 0.79).

#### Psychological Health and Metacognition Functioning

**(1) Behavior and Symptom Identification Scale**

The Italian version of this scale (Eisen et al., 1986; Conti, 1999) was used to assess the psychological health of subjects perceived during the antecedent week. Notably, 32 items clustered in five subscales compose the following: depression and anxiety, relation to self and others, psychosis, impulsive and addictive behavior, daily living and role functioning, and the overall score. The scale showed good reliability (α = 0.94).

**(2) The Metacognition Self-Assessment Scale**

The MSAS (Pedone et al., 2017) was used to assess metacognitive functions (e.g., “I'm able to define and detect my emotions”) according to the MMFM model (Semerari et al., 2003). The MSAS is scored using a five-point Likert scale (1 = never, 2 = rarely, 3 = sometimes, 4 = frequently, and 5 = almost always). The range of the total score is from 18 to 90. High scores on the MSAS indicate better self-evaluation of metacognitive abilities than low scores. The MSAS is designed to measure five sub-functions of metacognition as follows: (1) monitoring; (2) differentiation; (3) integration; (4) decentration; and (5) mastery. The total score is obtained from the sum of the five subscale scores, and this represents the overall level of metacognitive functioning. The scale showed good reliability (α = 0.91).

## Results

Table 1 reports zero-order Pearson correlations among the relevant variables of the study. In particular, we included age and gender because they were used as controls in subsequent analysis; variables in columns 3–7 correspond to facets of Behavior and Symptom Identification Scale (BASIS-32); and metacognition and all the unusual experience scales were used in the study.

**Table 1:** Zero-order correlations and descriptive statistics.

	**1**	**2**	**3**	**4**	**5**	**6**	**7**	**8**	**9**	**10**	**11**	**12**
1. Age	1											
2. Gender	0.06	1										
3. Relationships self/other	0.02	0.03	1									
4 Anxiety/depression	0.01	0.07	0.74^**^	1								
5. Daily living/role functioning	−0.00	−0.01	0.79^**^	0.77^**^	1							
6. Impulsive/addictive behavior	−0.01	−0.01	0.64^**^	0.63^**^	0.64^**^	1						
7. Psychosis	0.08	0.03	0.48^**^	0.61^**^	0.55^**^	0.74^**^	1					
8. Metacognition	−0.13	−0.00	−0.09	−0.07	−0.07	−0.13	−0.09	1				
9. RHS	0.02	−0.05	0.20^**^	0.28^**^	0.24^**^	0.36^**^	0.27^**^	−0.16^*^	1			
10. PDI	−0.13	−0.05	0.19^**^	0.24^**^	0.24^**^	0.35^**^	0.29^**^	−0.17^*^	0.52^**^	1		
11. LSHS	0.04	0.04	0.25^**^	0.32^**^	0.28^**^	0.33^**^	0.25^**^	−0.14^*^	0.54^**^	0.41^**^	1	
12. PQB	0.16^*^	0.05	0.34^**^	0.46^**^	0.40^**^	0.50^**^	0.42^**^	−0.29^**^	0.64^**^	0.70^**^	0.61^**^	1
Means (SD)	32.7 (9.6)	-	0.73 (0.69)	0.65 (0.69)	0.69 (0.62)	0.30 (0.43)	0.19 (0.38)	3.9 (0.59)	1.2 (0.31)	4.9 (3.2)	0.55 (0.57)	3.8 (3.3)
Females Means (SD)	32.29 (9.85)	-	0.71 (0.69)	0.61 (0.63)	0.69 (0.61)	0.30 (0.43)	0.18 (0.38)	3.9 (0.61)	1.26 (0.31)	5.06 (3.2)	0.53 (0.54)	3.66 (3.27)
Makes Means (SD)	33.51 (9.17)	-	0.76 (0.73)	0.72 (0.73)	0.68 (0.64)	0.29 (0.43)	0.21 (0.39)	3.9 (0.54)	1.22 (0.32)	4.70 (3.30)	0.58 (0.63)	4.03 (3.4)

*N = 207;*

* *p < 0.05;*

** *p < 0.01;*

*Gender (1 = F; 2 = M).*

*RHS, Revised Hallucination Scale; PDI, Peters Delusions Inventory; LSHS, Launay-Slade Hallucinations Scale; PQ-B, Prodromal Questionnaire-Brief*.

Since all the variables concerning unusual experiences were highly correlated, we run a principal component analysis. Eigenvalues were 2.722, 0.596, 0.437, and 0.245. This pattern clearly conformed to a one-component solution. All variables loaded strongly on the first component (range: 0.77–0.90). In the following analysis, we used the factor score deriving from the principal component analysis as an index of “Unusual Experiences.”

To investigate the associations of “Unusual Experiences” with its putative predictors, we run a series of hierarchical regression models. In Model 1, we entered age and gender as socio-demographic controls. In Model 2, we added the dimensions of BASIS 32. In Model 3, we added metacognition functioning. Table 2 summarizes the results.

**Table 2:** Hierarchical regression Models' fit indexes and standardized coefficients.

	**Model 1**	**Model 2**	**Model 3**
Gender	−0.01	−0.01	−0.01
Age	0.07	0.07	0.05
Relationships self/other		−0.21	−0.21
Anxiety/Depression		0.25^*^	0.25^*^
Daily living/role functioning		0.06	0.07
Impulsive/addictive behavior		0.43^**^	0.41^**^
Psychosis		−0.01	−0.02
Metacognition functioning			−0.16^**^
F(gdl)	0.45 (2,204)	10.28 (7,199)	10.14 (8,198)
R^2^	0.004	0.266^**^	0.291^**^
ΔR^2^		0.261^**^	0.025^**^
F change		(5,199) 14.157^**^	(1,198) 6,982^*^

* *p < 0.05;*

** *p < 0.01*.

Demographic control did not relate to “Unusual Experiences.” Entering the facets of BASIS32 significantly increased *R*^2^. Finally, metacognition added a further significant increase in *R*^2^.

Anxiety/depression and impulsive/addictive behavior showed positive and significant regression coefficients. Metacognition functioning showed a negative and significant regression coefficient. The coefficients for anxiety/depression and impulsive/addictive behavior remained significant.

## Discussion

The aim of this study was to extend the knowledge about the relationship between metacognition, psychopathology, and PLEs, redirecting the focus on metacognitive functioning rather than on metacognitive contents. Considering the existing evidence (Larøi and Van Der Linden, 2005; Stirling et al., 2007; Sellers et al., 2017), the levels of metacognitive abilities are expected to predict the outcome of PLEs in our sample, which includes non-clinical subjects.

As expected, the different psychopathological features were strongly correlated with the whole range of PLEs, in line with previous findings (Freeman and Fowler, 2009; Armando et al., 2010; Kelleher and Cannon, 2011; Varghese et al., 2011). Moreover, the specific PLEs turned out to be strongly interrelated as well, and this result allows us to speculate on the co-occurrence of such phenomena which has already been explained in the literature (Pechey and Halligan, 2011). Thus, a principal component analysis was run to pool the whole set of PLEs into a single factor to conduct the subsequent analyses. Concerning metacognition, from correlational analyses, it emerged that it was not related to any of the psychopathological domains explored, but it was significantly and negatively correlated with all the PLE scales, suggesting a potential role of metacognition.

To clarify these relationships, a hierarchical regression was performed, which showed the result that, as expected, age and gender did not predict PLEs (step 1), the subscales, namely, anxiety/depression and impulsive/addictive behavior, significantly predicted PLEs (step 2), and that metacognitive functioning significantly explained a further portion of variance after accounting for psychopathology (step 3).

The role of the subscales related to emotional and impulsive symptoms is in line with previous studies on the topic: a long tradition of research suggests a direct involvement of emotional features in the onset of hallucinations (Slade and Bentall, 1988), and recent empirical findings confirmed and clarified that high levels of anxiety constitute a remarkable vulnerability factor for PLE predisposition in non-clinical individuals as well as depression and stress (Freeman and Garety, 2003; Johns et al., 2004; Allen et al., 2005). Concerning *impulsive/addictive behavior*, results can be explained in light of the evidence regarding the relationship between substance addiction and PLEs (Mitchell and Vierkant, 1991; Rössler et al., 2007; Brewer and Collins, 2014).

Regarding step 3 of the hierarchical regression, it is possible to notice that metacognition negatively and significantly predicts PLEs conjunctly with psychopathological factors suggesting that metacognitive functioning does not overlap with psychopathological variables; however, the percentage of variance explained by metacognition is lower than the percentage explained by psychopathological variables. This result enlarges the present knowledge stemmed from the studies previously conducted (Larøi and Van Der Linden, 2005; Stirling et al., 2007; Sellers et al., 2017) measuring metacognition through the use of MCQ, which includes, among its factors, components related to the emotional sphere (e.g., worry and rumination). The use of MSAS allows us to assert that metacognitive functioning acts as an independent factor in predicting PLEs. Metacognitive abilities seem to play a protective role in the occurrence of PLEs among non-clinical individuals, and thus, it is possible to hypothesize that a good metacognitive functioning (that implies a balanced combination of different metacognitive distinct functions) allows for the interpretation of inner mental events and outer events to provide an explanation of the reality, which prevents the individual from experiencing PLEs.

## Conclusion

This study delivers a new inspiring perspective on the complex interplay between psychopathology, metacognition, and PLEs, suggesting that poor metacognitive functioning predisposes to the occurrence of PLEs in individuals, and such ability operates as an independent predictor along with other variables. Although rigorously conducted, this study is not exempt from limits. First, the study adopted a cross-sectional design, preventing us from making further inferences on the causal relationships between the variables observed. It is desirable that future research would extend the present findings throughout longitudinal studies to confirm the protective role of metacognitive functioning on the onset and development of PLEs. To have a comparative value between metacognitive functioning and metacognitive contents and to verify what already showed in previous literature, the MCQ questionnaire could have been administered adopting a different study design. Another limit may be constituted by the adoption of self-report questionnaires that present a desirability bias: it would be interesting in the future to assess metacognition through an interview that provides more detailed information on the peculiar metacognitive functioning of the individual. Finally, the obtained sample size is of only moderate magnitude. Therefore, the generability of results may turn out to be limited, and power could be inadequate for small population effect sizes.

## Data Availability Statement

The raw data supporting the conclusions of this article will be made available by the authors, without undue reservation.

## Ethics Statement

Ethical review and approval was not required for the study on human participants in accordance with the local legislation and institutional requirements. The patients/participants provided their written informed consent to participate in this study.

## Author Contributions

GN, AC, EB, MG, and CC: conceptualization. FF, MG, and CC: methodology. EB: formal analysis and visualization. MG: investigation. MD, FF, RR, CA, ED, and ME: resources. MG, FF, and EB: data curation. MG, CC, and EB: writing—original draft preparation. CA: writing—review and editing. AC and GN: supervision. EB and MG: project administration. All authors have read and agreed to the published version of the manuscript.

## Conflict of Interest

The authors declare that the research was conducted in the absence of any commercial or financial relationships that could be construed as a potential conflict of interest.

## Publisher's Note

All claims expressed in this article are solely those of the authors and do not necessarily represent those of their affiliated organizations, or those of the publisher, the editors and the reviewers. Any product that may be evaluated in this article, or claim that may be made by its manufacturer, is not guaranteed or endorsed by the publisher.
